# Observational Evidence for Desert Amplification Using Multiple Satellite Datasets

**DOI:** 10.1038/s41598-017-02064-w

**Published:** 2017-05-17

**Authors:** Nan Wei, Liming Zhou, Yongjiu Dai, Geng Xia, Wenjian Hua

**Affiliations:** 1 School of Atmospheric Sciences, Sun Yat-Sen University, Guangzhou, 519082 China; 2 Department of Atmospheric and Environmental Sciences, University at Albany, State University of New York, Albany, NY 12222 USA; 3 Key Laboratory of Meteorological Disaster, Ministry of Education (KLME)/Joint International Research Laboratory of Climate and Environment Change (ILCEC)/Collaborative Innovation Center on Forecast and Evaluation of Meteorological Disasters (CIC-FEMD), Nanjing University of Information Science & Technology, Nanjing, 210044 China

## Abstract

Desert amplification identified in recent studies has large uncertainties due to data paucity over remote deserts. Here we present observational evidence using multiple satellite-derived datasets that desert amplification is a real large-scale pattern of warming mode in near surface and low-tropospheric temperatures. Trend analyses of three long-term temperature products consistently confirm that near-surface warming is generally strongest over the driest climate regions and this spatial pattern of warming maximizes near the surface, gradually decays with height, and disappears in the upper troposphere. Short-term anomaly analyses show a strong spatial and temporal coupling of changes in temperatures, water vapor and downward longwave radiation (DLR), indicating that the large increase in DLR drives primarily near surface warming and is tightly associated with increasing water vapor over deserts. Atmospheric soundings of temperature and water vapor anomalies support the results of the long-term temperature trend analysis and suggest that desert amplification is due to comparable warming and moistening effects of the troposphere. Likely, desert amplification results from the strongest water vapor feedbacks near the surface over the driest deserts, where the air is very sensitive to changes in water vapor and thus efficient in enhancing the longwave greenhouse effect in a warming climate.

## Introduction

Changes in atmospheric composition due to elevated greenhouse gases (GHGs) modify the Earth energy budget and thus lead to various climate changes at global scales^1^. Such changes are not spatially uniform^2, 3^. Stronger warming is seen over land than oceans and in higher latitudes^4^, primarily due to spatial differences in GHGs induced radiative forcings and associated climate feedbacks^5^. The strong warming amplification over the Arctic, known as polar amplification^6^, is a typical example, which has been well recognized and extensively studied^1, 7, 8^.

Recent studies have identified another warming pattern that land surface air temperature (LSAT) in mid- and low- latitudes is amplified over deserts, referred to as desert amplification. By analyzing observational, reanalysis and simulated LSAT trends between 50°S–50°N for the period 1979–2012, Zhou *et al*.^9, 10^ showed that the warming rates in LSAT increased exponentially with the dryness of ecosystems and maximized over the driest ecoregions such as the Sahara desert and the Arabian Peninsula. Cook and Vizy^11, 12^ also found that the LSAT warming over the Sahara desert for 1979–2012 was 2–4 times stronger than that of the whole tropical areas using five reanalysis products and three observational temperature datasets, and that while there is amplified warming all months of the year over the Sahara, the amplification is greater during July- October. Zhou^13^ further examined the LSAT changes in historical and projected simulations for the period 1950–2099 and pointed out that desert amplification reflected a fundamental large-scale global warming pattern, not short-term temperature variability.

Mechanisms for desert amplification are not well understood. LSAT warms differently among ecoregions depending largely on surface radiative forcing and its partitioning between sensible and latent heat fluxes^14–16^. Instinctively, one would link the spatial differences in warming to different degrees of evaporative cooling effects induced by evapotranspiration (ET)^9, 17–19^. For a given radiative forcing, the surface warms less over wetter regions with more soil moisture as most of the forcing is turned into latent heat via ET. On the contrary, over deserts where soil moisture and thus ET are very limited, the surface tends to warm much faster because the forcing is mostly balanced by sensible heat and longwave radiation^10–13^. After analyzing the surface energy budget, Cook and Vizy^11, 12^ attributed the amplified warming over the Sahara desert primarily to the strong longwave radiative coupling between the surface and the atmosphere in response to increasing GHGs, and the seasonal variation of the amplified magnitude to the atmospheric moisture change associated with particular circulation system anomalies over the Sahara desert. Guan *et al*.^20^ found that the regional-scale enhanced warming over the drylands over East Asia were more driven by radiatively forced temperature changes than dynamically induced temperature changes. Zhou *et al*.^10^ and Zhou^13^ attributed desert amplification mostly to the enhanced downward longwave radiation (DLR) at the surface associated with stronger water vapor feedbacks over drier ecoregions in a warming climate.

Understanding how climate change over arid and semi-arid regions has important societal and economic implications in the context of anthropogenic global warming^13, 21, 22^. However, there are several caveats in the above detection and attribution analyses of desert amplification. First, deserts have very limited weather station records available for data collection, assimilation or model validation. This data gap limits the quality of *in-situ* derived global gridded observations, reanalysis products and climate model simulations, and thus could not provide reliable climate data over deserts. Second, regional and global climate models tend to have systematic biases in warm and dry climates and many current models overestimate regional amplification of global warming^23–25^. Third, the historical changes of observing system cause unexpected inhomogeneities in reanalyses, which likely compromise the long-term climate trend estimate in energy and hydrological cycles^26–28^. In particular, the previous attribution analyses have often used water vapor, DLR and other variables in surface energy budget from reanalyses and model simulations^10–13^, which are not well assimilated or validated over deserts due to data paucity. These caveats cast doubt on the existence and physical mechanisms of desert amplification.

To further examine desert amplification, here we analyze LSAT and troposphere temperature trends using long-term observations from multiple satellite-derived datasets for the period 1979–2015. Satellites allow global coverage by measuring the radiance of the Earth and thus can provide observations over data-paucity regions such as remote deserts. In addition, the linkages between temperature, DLR and water vapor are also analyzed using other short-term satellite products for the period 2003–2015. These 13-year satellite products serve as a compromise because there are no reliable long-term observed DLR and water vapor at global scales^1^. However, the water vapor response to a climate fluctuation at short-term scales should be about the same as that at long-term scales^29^, and thus these short-term datasets can be considered appropriate for use to further explore the physical mechanisms of desert amplification.

This study focuses only on the land areas between 50°S–50°N. It consists of a long-term temperature trend analysis and a short-term anomaly analysis of atmospheric temperatures, water vapor, and DLR (see Data and Method for details). For the long-term trend analysis, we use two Microwave Sounding Unit (MSU) based satellite data developed by the Remote Sensing System (RSS) analysis^30, 31^ and by the University of Alabama at Huntsville (UAH)^32^ and one satellite-station merged LSAT dataset by the NASA Goddard Institute for Space Studies (GISS)^4^ to examine desert amplification. Among the quantities in the MSU-based products, the temperature centered in the lower troposphere (termed TLT, about ~2 km above the surface) is our main concern as it represents the temperature properties of the atmosphere nearest the surface. The trends of temperatures covering the mid- to lower troposphere (termed TMT, centered about ~4 km) and mid- to upper troposphere (termed TTS, centered about ~10 km) are also plotted to show the vertical evolution of warming patterns and analyze the uncertainties in MSU-derived products. We use a satellite-gauge estimated precipitation dataset to approximately define the geographical distribution of surface dryness. To quantify how the warming rates vary by large-scale climatic zones as done previously^9, 10, 13^, the spatial patterns of temperature trends are depicted as a function of climatological precipitation in terms of 6, 12, 18 climate zones. As the essential features of desert amplification remain robust across all climate-zone classifications and all seasons, we focus only on the annual mean values and primarily show the results for the classification of 12 climate zones in most cases for simplicity. For the short-term anomaly analysis, our main focus is the spatial and temporal coupling of the changes among temperatures, DLR and water vapor. The satellite-derived sounding data provide not only LSAT and total atmospheric water vapor content (TWV), but also pressure-stratified tropospheric temperatures (T) and specific humidity (*q*). High-quality satellite retrievals of surface DLR and its corresponding Cloud Radiative Effect (DLRCRE) as well as surface downward solar radiation (DSR) are also obtained. We first identify the particular significant fluctuation of LSAT in 2010 relative to its climatology (excluding 2010) and examine the overall coupling of LSAT, DLR and TWV anomalies, and then compare the vertical profiles of T and *q* anomalies in 2010 over two extreme climate zones (the driest and wettest from the 12 large-scale climate zones) as well as their associations with DLR and LSAT. These analyses help to assess the relative contributions of atmospheric T and *q* changes to surface warming among different climate zones and provide further insight into the mechanisms of desert amplification.

## Long-term trend analysis for desert amplification

**Figure 1:**
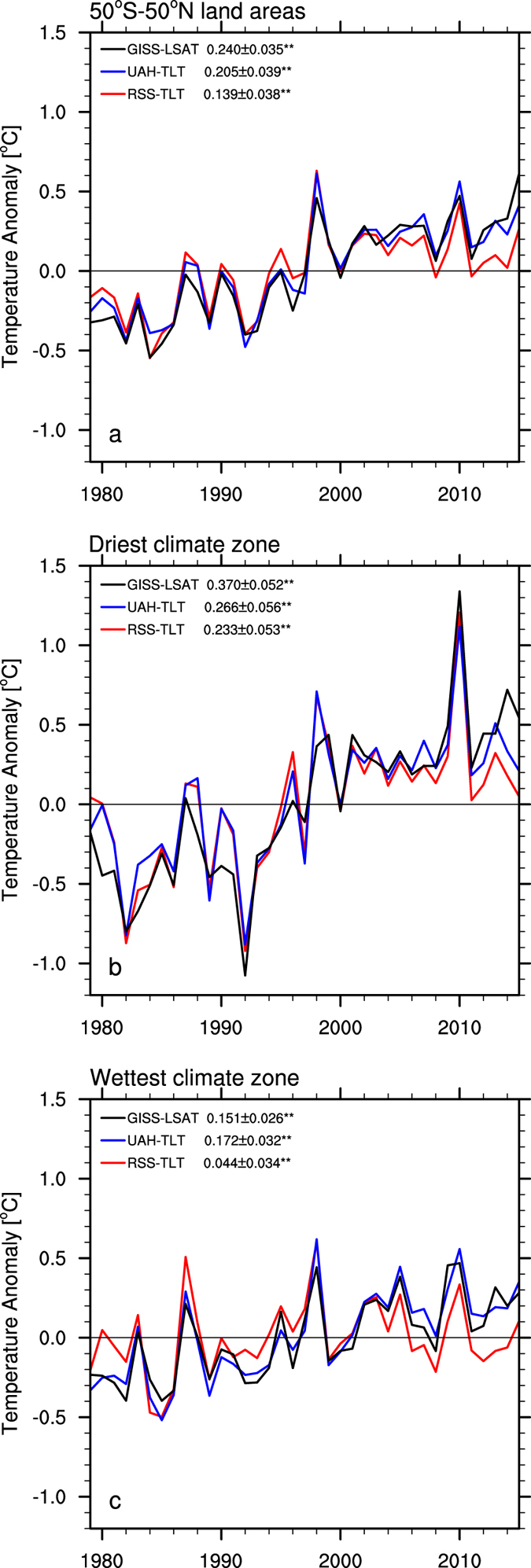
Zonal mean annual temperature anomalies (°C) for the period 1979–2015 from RSS-TLT, UAH-TLT and GISS-LSAT averaged over (**a**) the entire study region, and over the (**b**) driest and (**c**) wettest from the 12 large-scale climate zones classified by precipitation. Linear trends (°C/decade) ± one standard deviation with ‘**’ are statistically significant (p < 0.05).

Figure 1a shows the zonal mean annual anomalies in TLT from RSS and UAH and LSAT from GISS for the period 1979–2015 averaged over the entire study region. The three temperatures exhibit similar inter-annual variabilities and significant warming trends (p < 0.05) ranging from 0.14 ± 0.038 to 0.24 ± 0.035 °C/decade, indicating that the dominant long-term global warming signal is well captured by all satellite measurements. LSAT has a relatively stronger warming magnitude than TLT. Figure 1b and c illustrate the zonal mean temperature anomalies between two extreme climate zones, the driest and wettest of the classified 12 large-scale climate zones by precipitation. Evidently, the former has stronger inter-annual variabilities and larger warming trends in the three datasets, ranging from 0.23 ± 0.05 to 0.37 ± 0.05 °C/decade, than the latter, ranging from 0.05 ± 0.03 to 0.17 ± 0.03 °C/decade. Note that all the trends are statistically significant (p < 0.05). In the driest zone, the three temperature anomalies all reach the historical warmest value in 2010, ranging from 1.12 to 1.36 °C.

**Figure 2:**
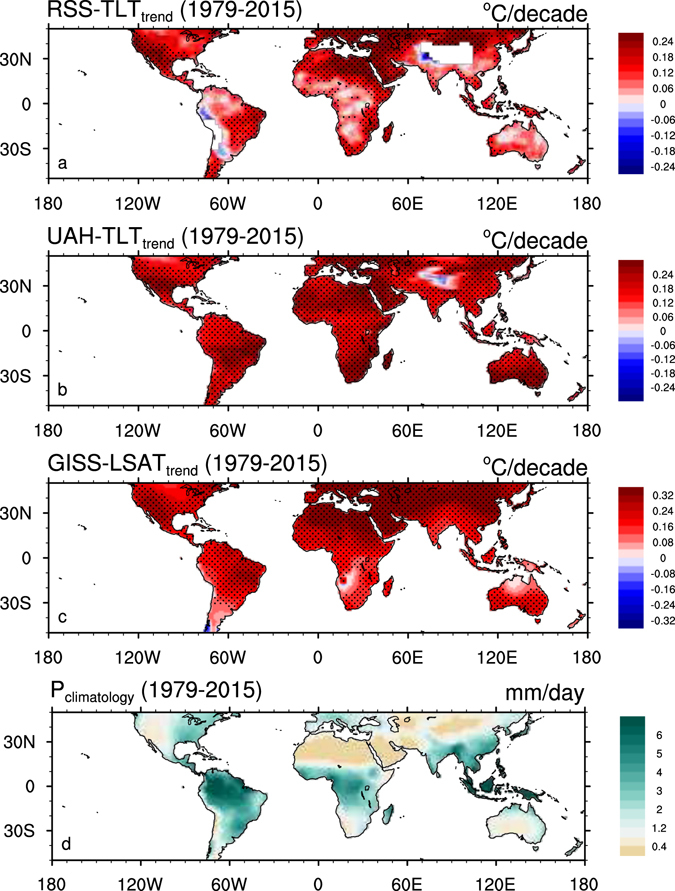
Spatial patterns of annual temperature trends (°C/decade) from (**a**) RSS-TLT, (**b**) UAH-TLT and (**c**) GISS-LSAT for the period 1979–2015 and (**d**) spatial patterns of climatological annual precipitation (mm/day) for the period 1979–2015, in 2.5° × 2.5° grid boxes over land. Stippling indicates regions where temperature trends are statistically significant (p < 0.1). Map was created using the NCAR Command Language (Version 6.0.0) [Software]. (2011). Boulder, Colorado: UCAR/NCAR/CISL/TDD. http://dx.doi.org/10.5065/D6WD3XH5.

The spatial patterns of annual temperature trends are given in Fig. 2, together with the climatology of annual precipitation. The temperatures increase almost everywhere except for parts of Australia, central Africa, central U.S. and northern Amazon in RSS-TLT. Among the three temperature datasets, the strongest warming trends consistently occur over the driest arid regions such as the Sahara desert and the Arabian Peninsula, Middle East, northern China and western U.S. This warming pattern is strongly correlated with the geographic distribution of climatological precipitation, with a spatial correlation ranging from −0.50 in RSS-TLT (p < 0.01, n = 1538, where n is the sample size) to −0.66 in GISS-LSAT (p < 0.01, n = 1538). Note that pronounced warming also appears over several non-arid regions such as southern Amazon, Europe and eastern U.S., possibly due to regional-scale changes in soil moisture, dynamical circulation, and sea surface temperature^10, 13, 33^.

**Figure 3:**
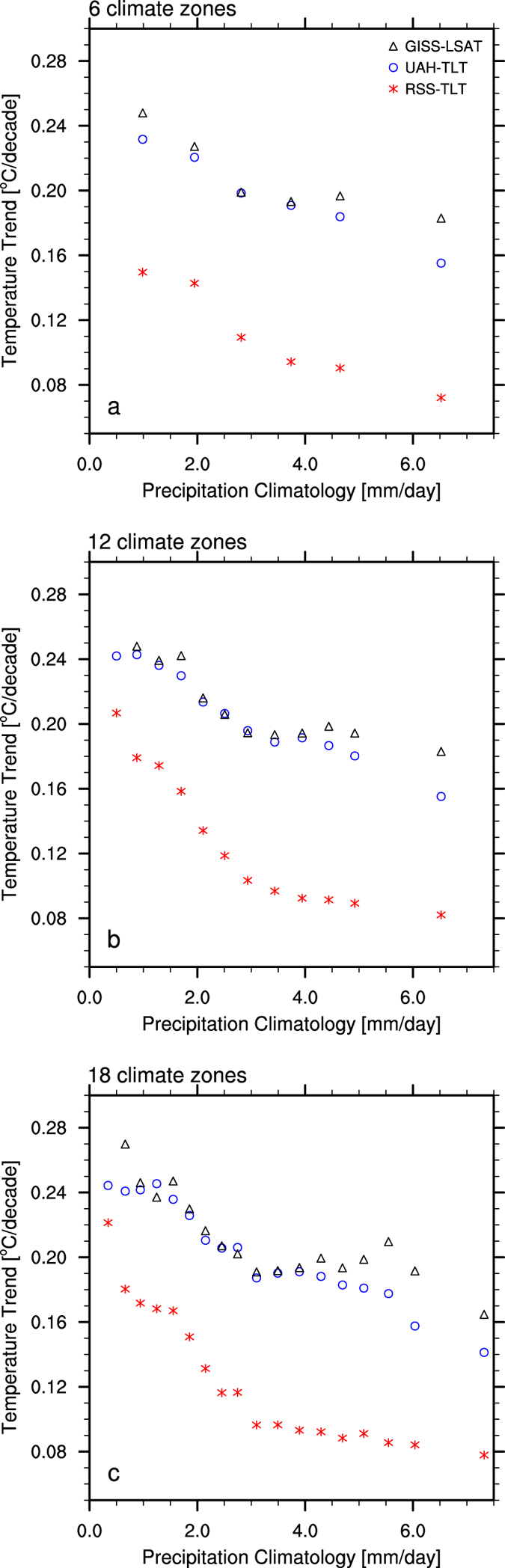
Zonal mean annual temperature trends (°C/decade) of RSS-TLT, UAH-TLT and GISS-LSAT for the period 1979–2015 as a function of zonal mean climatological annual precipitation (mm/day) in terms of (**a**) 6, (**b**) 12 and (**c**) 18 large-scale climate zones.

To maximize large-scale warming patterns and minimize regional- and local- scale temperature variabilities, Fig. 3 displays the annual trends of TLT and LSAT as a function of surface dryness in terms of 6, 12 and 18 climate zones classified by climatological precipitation. Evidently, the warming rates increase dramatically with the decrease of precipitation amount, with the largest values occurring over the driest regions. This pattern is consistent for both TLT and LSAT across all climate-zone classifications, indicating a strong spatial dependence of satellite-derived warming patterns on surface dryness. UAH-TLT and GISS-LSAT exhibit similar features, while the warming magnitude in RSS-TLT is relatively small, likely due to the missing data over high-altitude areas and the presence of insignificant warmings over parts of Australia, central Africa, central U.S. and northern Amazon as discussed previously. Overall, the satellite-derived products consistently confirm that near-surface warming is generally strongest over the driest regions, in agreement with the major features of desert amplification^9–11, 13^.

**Figure 4:**
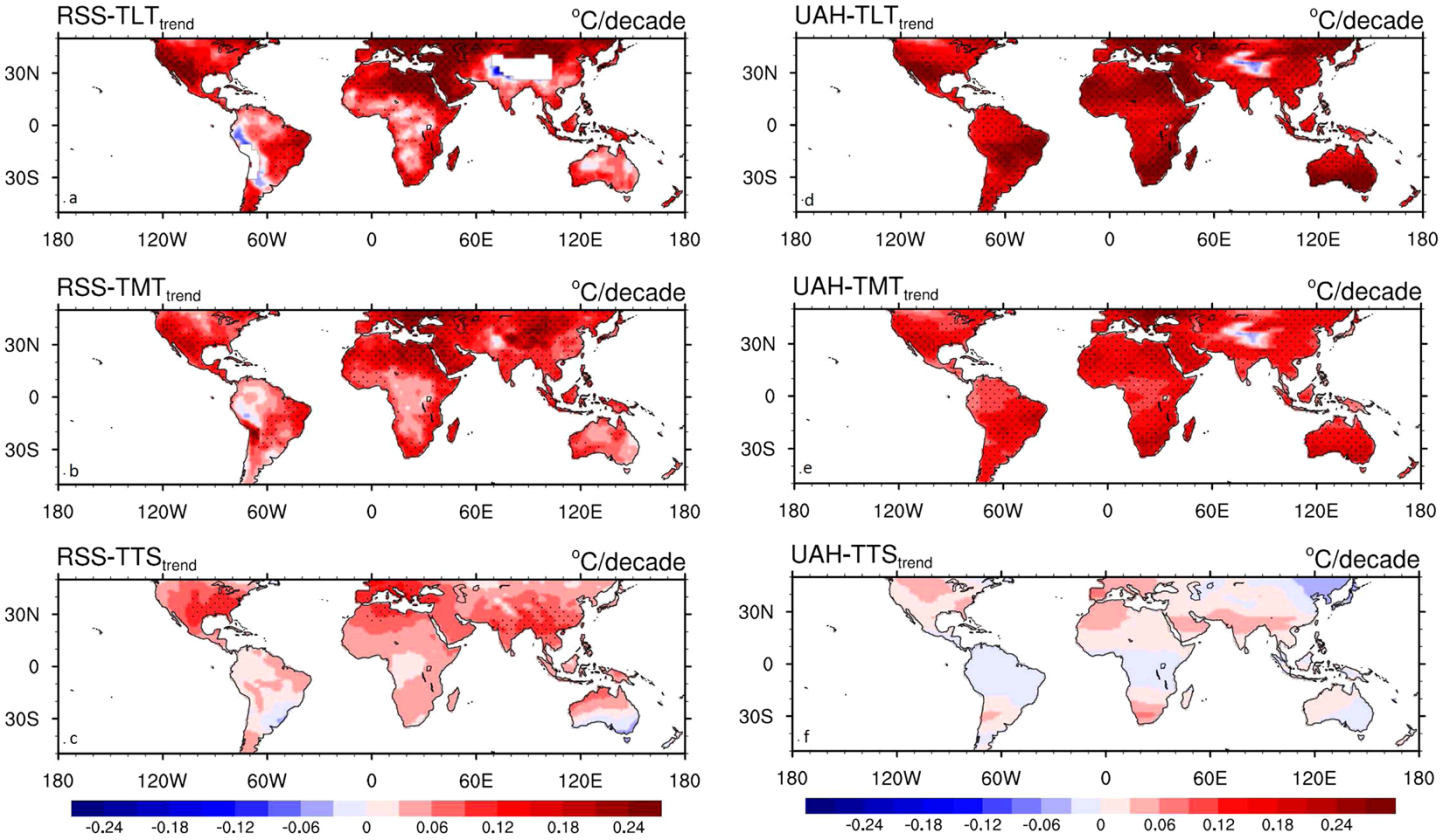
Spatial patterns of annual temperature trends (°C/decade) from (**a**) RSS-TLT, (**b**) RSS-TMT, (**c**) RSS-TTS, (**d**) UAH-TLT, (**e**) UAH-TMT and (**f**) UAH-TTS for the period 1979–2015, in 2.5° × 2.5° grid boxes over land. Stippling indicates regions where temperature trends are statistically significant (p < 0.1). Map was created using the NCAR Command Language (Version 6.0.0) [Software]. (2011). Boulder, Colorado: UCAR/NCAR/CISL/TDD. http://dx.doi.org/10.5065/D6WD3XH5.

Next we examine the vertical features of desert amplification by comparing the warming patterns among the three tropospheric temperature quantities, namely TLT, TMT and TTS, from RSS and UAH. Figure 4 shows a weakening of desert warming amplification with altitude from the lower to upper troposphere and the warming rate in TTS is almost spatially independent from surface dryness. The spatial dependence of temperature trends on precipitation by large-scale climate zone shows similar features (Figure S1), with the desert amplification pattern to be strongest in TLT, followed by TMT and the weakest in TTS. Again, these features change little under different climate-zone classifications and thus only the results from the classified 12 climate zones are displayed.

Despite the differences in magnitude among different satellite data, our long-term trend analyses of the satellite-derived temperatures have confirmed the existence of warming amplification over deserts in near surface and the lower troposphere (LT). Desert amplification is strongest near the surface, gradually decays with height, and mostly disappears in the upper troposphere (UT). Such vertical evolution is very consistent with the findings from reanalysis data in Cook and Vizy^11^. These results indicate that desert amplification is a real large-scale warming pattern, not an artifact due to scarcity of ground-based observations.

## Short-term anomaly analysis for the coupling between LSAT, DLR and TWV

Previous studies have proposed that desert amplification may be attributable to GHGs-induced DLR increases associated with stronger water vapor feedbacks over drier ecoregions^10, 13^. Next we use the 13-year satellite-derived sounding data to further this linkage among atmospheric temperatures, DLR and water vapor for attributing desert amplification. The features of desert amplification are highlighted by comparing the results between the driest and wettest of the classified 12 large-scale climate zones as detailed below. Consistent results are also obtained under different climate-zone classifications, which are not shown for brevity.

**Figure 5:**
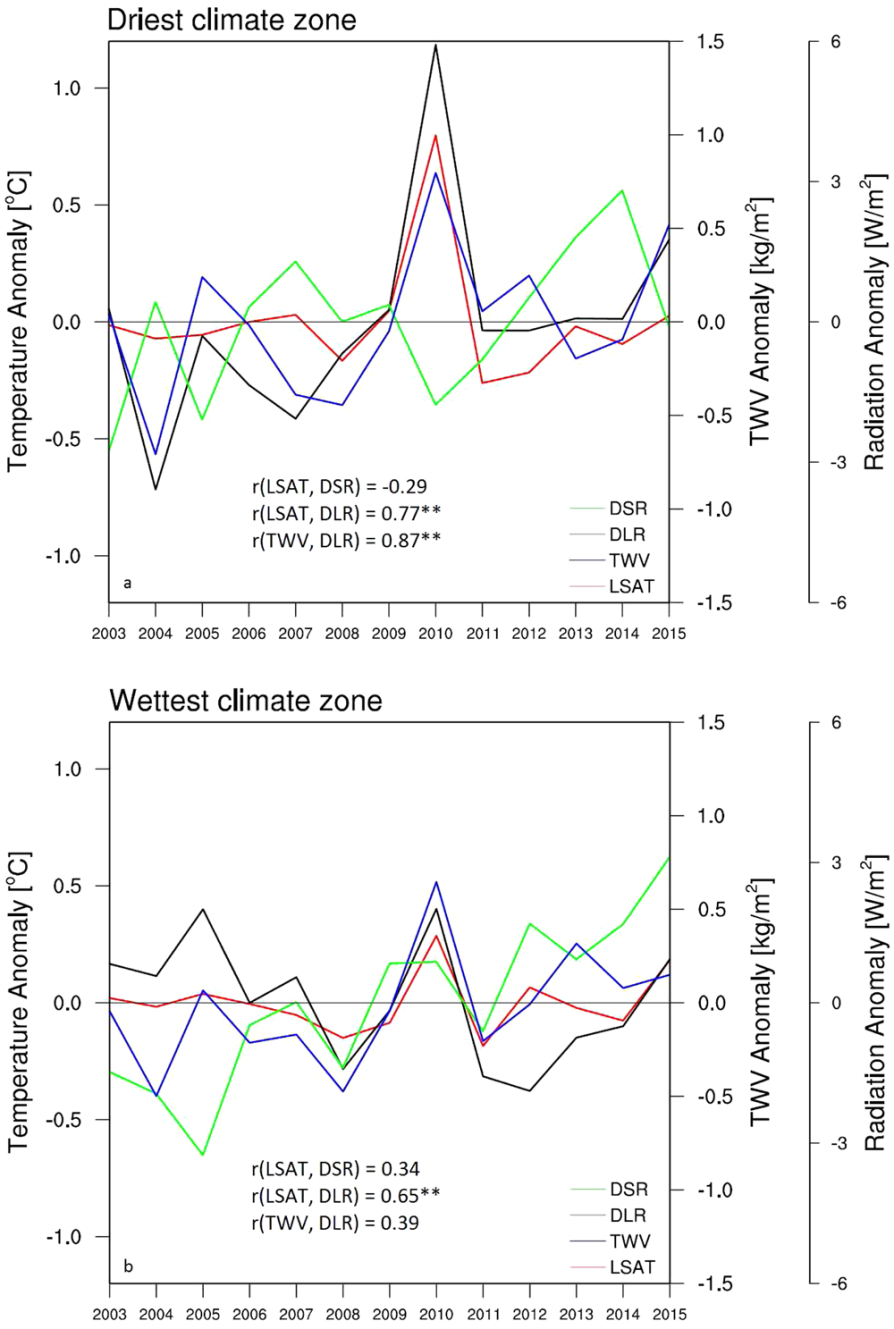
Zonal mean anomalies of LSAT (°C) and TWV (kg/m^2^) from AIRS and DSR (W/m^2^) and DLR (W/m^2^) from CERES-EBAF for the period 2003–2015 averaged over the (**a**) driest and (**b**) wettest from the 12 large-scale climate zones classified by precipitation. Correlation coefficients with “**” are statistically significant (p < 0.05).

The warming rate of LSAT depends on the magnitude of surface radiative forcings. Figure 5 shows the zonal mean annual anomalies of LSAT, DLR and TWV for the period 2003–2015 over the driest and wettest climate zones. For the driest zone, the three variables share very similar inter-annual variability, with a strong correlation of 0.77 (p < 0.05) between LSAT and DLR, and 0.87 (p < 0.05) between DLR and TWV, indicating a close coupling among LSAT, DLR and TWV changes. For the wettest zone, the three variables share a weaker covariance. DLR has a correlation of 0.65 (p < 0.05) with LSAT and 0.39 with TWV. DSR anomalies exhibit no or little correlations with LSAT or DLR in both zones. These results indicate that the increase in DLR is the major radiative forcing for the surface warming, while the changes in DSR has little impact. The much larger correlations of LSAT with DLR and TWV over the drier climate indicate a stronger sensitivity of LSAT to changes in DLR and water vapor over deserts.

It is notable that LSAT varies slightly for 2003–2015, except 2010 when there is a significant warming fluctuation over the two extreme climate zones (Fig. 5), particularly in the driest climate zone where this fluctuation represents the hottest year from the long-term temperature records (Fig. 1). Correspondingly, there is also a similarly significant increase of DLR and TWV (Fig. 5). The DLR anomaly in 2010 reaches 6.0 W/m^2^ over the driest zone and is 2.0 W/m^2^ over the wettest zone. It is likely that the strong El Niño event in the first half year of 2010 plays a key role in this positive fluctuation^29, 34^, along with contributions from short time-scale weather variability^35^. This fluctuation can serve as a good case study to validate the currently proposed mechanisms of desert amplification. We will test whether the significant warming in 2010 is spatially coupled with the enhanced DLR in response to a warming and thus moistening atmosphere, and whether such coupling is strongest over deserts.

**Figure 6:**
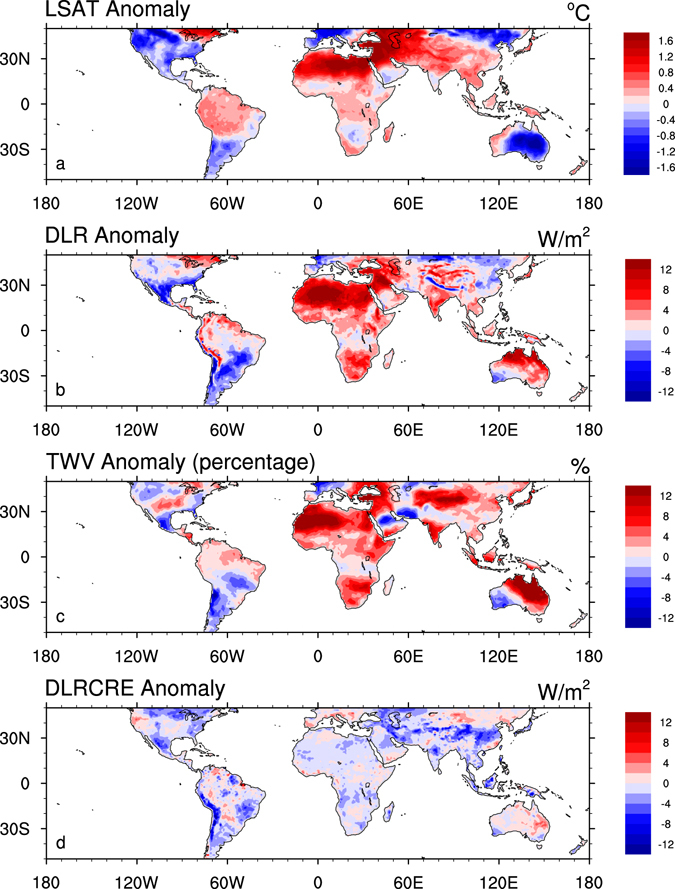
Spatial patterns of annual anomalies in 2010 for (**a**) LSAT (°C), (**b**) DLR (W/m^2^), (**c**) TWV (%) and (**d**) DLRCRE (W/m^2^), in 1° × 1° grid boxes over land. The anomalies are calculated as the difference in the annual averages in 2010 and the climatology aggregated from other years (2003–2009 & 2011–2015). For the TWV plot, the anomaly is expressed as the percentage divided by the climatological TWV value. Map was created using the NCAR Command Language (Version 6.0.0) [Software]. (2011). Boulder, Colorado: UCAR/NCAR/CISL/TDD. http://dx.doi.org/10.5065/D6WD3XH5.

If the surface warming is primarily attributable to the increase in DLR, one would expect to see a spatial coupling between them. Figure 6 displays the spatial patterns of LSAT and DLR anomalies in 2010. As expected, the largest LSAT increases occur mainly over the arid ecosystems such as the Sahara desert and parts of Western and Central Asia (Fig. 6a) where DLR increases most (Fig. 6b), indicating DLR is a major driver of surface warming. Similarly, if the increase in DLR is at least partially attributable to the increase in TWV, one would expect to see a spatial coupling between them as well. Evidently, the fractional increases in TWV show similar patterns to the increases in LSAT and DLR (Fig. 6c). Note that it is the fractional change in water vapor instead of the absolute value that governs the changes in DLR due to the logarithmic function of DLR on water vapor concentration^16^. The driest climate zones have the least amount but the largest fractional increases in TWV, and consequently the greatest efficiency in increasing DLR and thus LSAT. In addition, cloud cover seems to have little contribution to the changes in DLR (Fig. 6d) even in the wettest climate regions as shown in Zhou^13^, likely due to the insensitivity of DLR to the TWV changes under hot and humid conditions^13, 36^.

Our short-term anomaly analysis above shows the strongest coupling among LSAT, DLR and TWV over the driest climate zones, indicating that DLR is the primary radiative forcing for LSAT changes over deserts. This inference is analogous with the long-term trend analyses from observational and reanalysis data^9–12^ and from historical and projected simulations^13^. These results confirm that desert amplification could be driven primarily by the increases in DLR, which are closely linked to the strongest water vapor feedbacks over the driest climate zones as discussed next.

## Water vapor feedbacks inferred from short-term climate anomalies in 2010

DLR is a vertical integral of downward longwave radiation of the entire atmosphere reaching the surface and depends on the vertical profiles of atmospheric humidity (*q*) and temperature (T). It generally has the most contribution from the LT, particularly near surface layers over hot and wet ecoregions^37^. As the increase in water vapor is closely linked to surface and tropospheric warming^16^, the enhancement of DLR results likely from a warming and thus moistening atmosphere^10, 13^. Previous studies have used short-term disturbances such as ENSO events to investigate water vapor feedbacks^29, 35^. Here we follow such ideas to infer whether the largest increase in DLR over deserts could be attributable to the strongest water vapor feedbacks over the driest regions.

**Figure 7:**
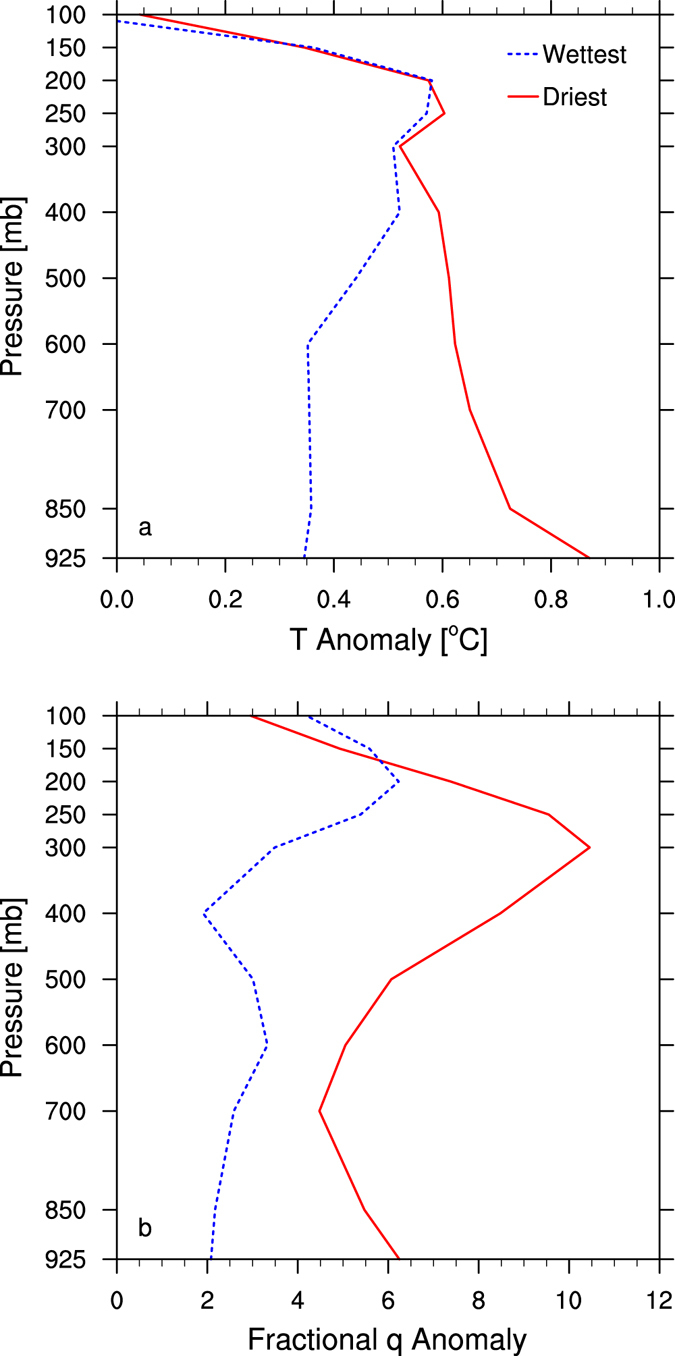
Zonal mean annual anomalies in 2010 for (**a**) T (°C) and (**b**) *q* (%) as a function of pressure (mb) over the driest and wettest from the 12 large-scale climate zones classified by precipitation. The anomalies are calculated as the difference in the annual averages in 2010 and the climatology aggregated from other years (2003–2009 & 2011–2015). For the *q* plot, the anomaly is expressed as the percentage divided by the climatological *q* value.

Figure 7 shows the vertical profiles of T and *q* anomalies in 2010 over the driest and wettest from the classified 12 large-scale climate zones. There is an overall warming up to 0.9 °C in T and an overall moistening up to 10% in *q* for the entire atmospheric layers below 100 mb. In the wettest zone, the warming enhances with height from the surface and maximizes in the UT around 200 mb (Fig. 7a), and a similar vertical pattern of moistening is also seen in *q* (Fig. 7b). This pattern is very consistent with the well-known water vapor feedback which maximizes in the tropical UT where the warming profile is close to moist adiabatic and the fractional changes in water vapor concentration are largest with increasing GHGs^16^. In the driest zone, there are two warming maxima, one in the UT around 250 mb and the other near the surface (Fig. 7a). The corresponding fractional changes in *q* also show two maxima, one around 300 mb and the other near the surface (Fig. 7b). Evidently the maximum UT warming in both climate zones is comparable and exhibits little spatial dependence on surface dryness, which is expected as large-scale circulation and wave propagation in the UT mostly reduce the spatial gradients in T and *q* profiles that are often observed in the LT^13^. However, from 400 mb downward, the warming contrast between the two zones enhances with decreasing altitude, with a slightly weakened warming over the wettest zone but a significantly intensified warming over the driest zone. As a result, the amplified warming over the driest zone maximizes near the surface. In correspondence, the water vapor also increases significantly near the surface over the driest region, where the air is very dry and thus sensitive to the change in atmospheric moisture. Overall the vertical profiles of warming correspond well to these of moistening in both climate zones, indicating that the warming and moistening of the atmosphere both enhances DLR and thus warms LSAT, particularly over deserts. It is worth noting that the vertical profiles in warming also display a weakening of desert amplification with altitude, which is consistent with the long-term temperature trend analysis from RSS and UAH datasets in Section 3.1. This weakening is reasonable as the changes in T and *q* tend to be spatially more uniform in the UT as discussed above.

**Figure 8:**
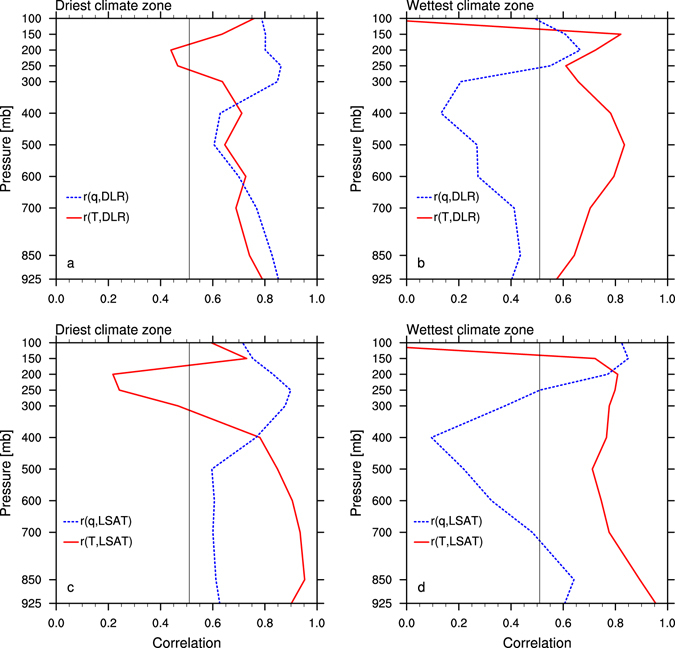
Correlations between atmospheric annual T and *q* anomalies with the annual anomalies of (**a**,**b**) DLR and (**c**,**d**) LSAT as a function of pressure (mb) for the period 2003–2015 averaged over the (**a**,**c**) driest and (**b**,**d**) wettest from the 12 large-scale climate zones classified by precipitation. Correlation coefficients greater than 0.51 (the black vertical line) are statistically significant (p < 0.05).

Next we simply use temporal correlations to quantify the relative contribution of atmospheric warming and moistening effects on DLR and LSAT (Fig. 8). T and *q* have a comparably strong correlation with DLR over the driest zone (Fig. 8a), while due to the aforementioned insensitivity of DLR to water vapor changes, only T strongly correlates with DLR over the wettest zone (Fig. 8b). Similar results are also obtained on the correlative relationship of T and *q* with LSAT given the close linkage between DLR and LSAT (Fig. 8c–d). Evidently the water vapor increases cannot occur without the temperature increases, and hence the largest increase in DLR and thus LSAT over deserts should be a consequence of the warming and thus moistening effect of the atmosphere.

Based on the above analyses together with previous studies^9–13^, we summarize the plausible mechanisms for desert amplification as follows. Two types of water vapor feedbacks are likely involved in desert amplification^13^. One is the water vapor feedback near the surface defined from the perspective of surface energy budget. The other is the widely recognized water vapor feedback which maximizes in UT defined from the perspective of Top of Atmosphere (TOA) radiation budget. For clarity, we refer to the former as the near surface water vapor feedback to differentiate it from the conventional water vapor feedback. For the case of the near surface water vapor feedback, deserts have the least water vapor content in the LT, particularly near the surface, and thus are most sensitive to changes in water vapor because it is fractional changes in water vapor that matters most in longwave absorption and emission^16^. In a warming climate, the warming and thus moistening atmosphere enhances DLR, which increases surface temperature more over drier regions due to lower ET; the warmer surface, in return, induces more upward longwave radiation into the atmosphere, which results in more absorption and thus more warming in the LT; the resulting warmer and thus moister atmosphere will trigger more DLR, which further amplifies the near-surface warming. This positive near surface water vapor feedback maximizes over the lowest atmosphere layers near deserts where the air is driest and thus most sensitive to the warming and moistening disturbances. In addition, it is also possible that the conventional water vapor feedback could have some contribution to the surface warming over the deserts as well due to less infrared opacity^13^. In such cases, the scarcity of water vapor and clouds over the deserts with least opaqueness to thermal infrared radiation can help propagate more enhanced UT thermal emission to reach and heat the surface. Our above statistical analysis indicates that the warming and moistening effects have comparable contributions to the DLR and LSAT increases over deserts. But quantitatively isolating the individual contribution of water vapor feedbacks from the UT, LT and near surface needs carefully designed sensitivity experiments with climate models, which will be done in future work.

## Uncertainties in satellite-derived data

The aforementioned analyses of both long-term and short-term satellite products indicate that desert amplification is confined to the LT, decays with height, and disappears in the UT. This is reasonable as the influence of land surface on the atmosphere decreases with altitude. On the other hand, in a warming climate the largest overall warming is expected to be seen in the tropical UT especially over deep convection regions where the warming profile should follow moist adiabat to be consistent with the strongest positive water vapor feedback and a corresponding negative lapse rate feedback as reflected in theoretical studies and climate simulations^1, 13, 16, 38–40^. This feature is supported by our short-term temperature anomaly analysis. However, the long-term trend analysis of UAH and RSS data shows a rapid decrease in warming from the LT to UT almost everywhere, with nearly no significant warming trends in TTS (Fig. 4; Figure S1). Other satellite data such as the recent product created by NOAA show similar patterns, with significantly weakened warming in the UT compared to that in the mid-troposphere^41^. These results demonstrate apparent discrepancies with the conventional view of water vapor/lapse rate feedbacks which can be principally controlled by the regional variations in surface temperature changes^1, 13, 16, 38–40, 42, 43^.

To our knowledges, two types of uncertainties in constructing temperature records from MSU-based radiance measurements are the possible sources for the discrepancies^43^. The first is the so-called structural uncertainties which originate from various adjustments to records for removing most of non-climatic signals induced by artificial or instrumental effects such as the inter-satellite calibration and orbital drift and decay^32, 39, 44^. These structural uncertainties produce considerable difficulties in generating homogenous climate records, which hampers a more realistic estimation of atmospheric temperature trends^42, 45^. The second is the componential uncertainties which are derived from stratospheric contamination on tropospheric temperature signals. The lower stratosphere is dominated by a strong cooling effect^1, 46^, which has been significantly included in TMT and TTS calculations and thus likely caused an underestimated tropospheric warming^42, 43, 47^. Overall, these two types of uncertainties in MSU-derived satellite data likely introduce spurious cooling signals to tropospheric temperature trends and thus weaken the warming effects throughout most of the troposphere.

## Conclusions

Recent studies have identified that the large-scale warming patterns of LSAT in the mid- and low- latitudes depends strongly on land surface dryness, with the largest warming rate occurring over the driest desert regions, namely desert amplification, and this amplification was attributed primarily to the strong longwave radiative coupling between the surface and the atmosphere^11, 12^ and to the enhanced DLR at the surface associated with stronger water vapor feedbacks over drier ecoregions^9, 10, 13^ in response to increasing GHGs. However, considerable uncertainties exist in observations, reanalyses and climate simulations over data-paucity deserts, which cast doubt on the detection and attribution of desert amplification. As satellite data have global coverage and can provide valid observations over remote deserts, here we further our understanding of desert amplification using multiple satellite-derived datasets over land between 50°S–50°N.

We first analyze the long-term temperature trends from RSS-TLT, UAH-TLT and GISS-LSAT datasets for the period 1979–2015 in terms of spatial patterns and large-scale climate zones. All satellite products consistently confirm that near-surface warming is generally strongest over the driest regions, in agreement with the pattern of desert amplification. We also examine the vertical evolution of desert amplification by comparing the warming patterns among TLT, TMT and TTS from the RSS and UAH datasets. Desert amplification is strongest near the surface, gradually decays with height, and mostly disappears in the UT, which is expected as surface dryness and large-scale circulation dominate the spatial patterns of warming in the lower and upper atmosphere, respectively^13^. These results indicate that desert amplification is a real large-scale warming pattern, not an artifact due to scarcity of ground-based observations.

Next we perform the short-term anomaly analysis of temperatures, water vapor and DLR from the satellite-derived sounding data for the period 2003–2015 to explore possible mechanisms for desert amplification. The desert amplification features are highlighted by comparing the results between the driest and wettest of the 12 large-scale climate zones classified by precipitation. First, the coupling among inter-annual variations in LSAT, DLR and TWV indicates that DLR is the primary radiative forcing for near surface warming and that LSAT is most sensitive to the changes in DLR and water vapor over the driest deserts. Second, the significant warming fluctuation in LSAT in 2010 is spatially coupled with the increases in DLR and TWV, and such coupling is strongest over deserts. This indicates again that the large increase in DLR is the major driver of near surface warming and is tightly associated with increasing TWV over deserts. Third, the vertical profiles of atmospheric temperatures and water vapor anomalies in 2010 show that desert amplification decays with altitude, consistent with the results of the long-term temperature trend analysis, and suggest that desert amplification is due to comparable warming and moistening effects of the troposphere. Therefore we infer that desert amplification results likely from the strongest water vapor feedback near the surface over the driest deserts, where the air is very dry and thus efficient in increasing longwave absorption and emission (i.e., the greenhouse effect).

Our results of both long-term and short-term satellite products consistently indicate that desert amplification is confined to the LT, decays with height, and nearly disappears in the UT, which is reasonable as surface dryness has smaller impacts on the above atmosphere in higher altitude. However, in a warming climate the largest warming effect is expected to be seen in the tropical UT especially over the wettest regions where water vapor has the strongest positive feedback^1, 16^. Although our short-term temperature anomaly analysis also supports this expectation, the long-term trend analysis of UAH and RSS data shows a widespread rapid decrease in warming from the LT to UT, with nearly no significant warming trends in TTS. This discrepancy may be due to some structural and componential uncertainties in the MSU-based satellite-derived temperatures, which likely induce spurious cooling signals to the tropospheric warming trends and thus underestimate the tropospheric warming strength.

The present work has made some unique contributions in understanding desert amplification. It provides the first observational evidence using multiple satellite-derived datasets that desert amplification is a real large-scale pattern of warming mode in near surface and low-tropospheric temperatures. In particular, the short-term anomaly analysis is used to explore the physical mechanisms of desert amplification based on the spatial and temporal coupling of the changes among temperatures, DLR and water vapor. In the previous studies, Cook and Vizy^11, 12^ showed the vertical dependence of warming trends using three reanalyses but could not identify conclusively water vapor effects due to reanalysis inhomogeneities; Zhou^13^ attributed desert amplification to the water vapor feedbacks using simulations from climate models but these models have systematic biases in warm and dry climates. Our analyses of the vertical atmospheric profiles deepen our understanding of the increases in DLR and LSAT over deserts and provide a quantitative basis for the relative contributions of the warming and moistening effects of the atmosphere to desert amplification.

Our analysis has focused on the primary thermodynamic mechanisms of desert amplification from the perspective of water vapor feedbacks as previous studies^9, 10, 13^ concluded that desert amplification reflects primarily the first order large-scale thermodynamic component of global warming. We do believe that atmospheric circulation plays an important role in warming and moistening the atmosphere over deserts via heat and moisture advection^12^. Admittedly, the enhanced DLR associated with the water vapor feedbacks is certainly not the only explanation for desert amplification. Other climate feedbacks and/or behaviors related to processes of land surface and/or planetary boundary layer may also have some contributions. Future work will include more feedbacks and processes into well-performed climate models to quantify their relative contributions and thus identify the determinant mechanisms for desert amplification.

## Data and Methods

### Long-term satellite-derived datasets

This study adopts the widely used satellite data developed by the Remote Sensing System (RSS) analysis^30, 31^ and by the University of Alabama at Huntsville (UAH)^32^ to examine the long-term warming trends between 1979–2015, with the latest versions RSSv3.3 and UAHv6beta4 analyzed. The two datasets are both derived from the (Advanced) Microwave Sounding Unit (MSU) instruments which measure thermal emissions at different frequencies, and hence a set of vertical-weighted averages of atmospheric temperatures peaking at different altitudes are provided. Among the published quantities, the temperature from MSU channel 4 mostly covers the lower stratosphere (termed TLS), with a theoretical cooling trend primarily caused by ozone depletion in recent decades^1, 46^. The MSU channels 2 and 3 mainly measure the temperatures of mid- to lower troposphere (termed TMT) and of mid- to upper troposphere (termed TTS), respectively, but they also contain the stratospheric contributions, with about 10–15% of TMT and more of TTS signals originating from the stratosphere^1^. There is another derived temperature of lower troposphere (termed TLT) which differs from the near-nadir and off-nadir scans of TMT. TLT has a weighting function centered in the lower troposphere, which is significantly lower than that in TMT^31, 48, 49^, and hence it has the least stratospheric contributions. Based on the vertical distributions of these temperatures, the TLT is our main concern because it is most appropriate to represent the temperature properties of the atmosphere near the surface. TMT and TTS trends are also plotted to show the vertical evolution of warming patterns and analyze the uncertainties in MSU-derived products. The two satellite products differ, particularly for TLT and TMT, in their methodologies of constructing continuous temperature records, including inter-satellite calibrations, corrections for diurnal drift and computations of gird-level averages from MSU channels^32, 39, 44, 50^. Nevertheless, we choose both here to demonstrate that our results are independent of one particular dataset. In addition, a gridded monthly LSAT dataset at the NASA *Goddard Institute for Space Studies* (*GISS*)^4^ is used to directly examine desert amplification near the surface. GISS temperatures are considered to be reliable over arid and semi-arid regions because surface observations are extensively merged with various satellite measurements^51^.

### Satellite-gauge merged precipitation data

In order to quantify whether atmospheric warming rates depend spatially on surface dryness, this study uses the advanced precipitation data from NOAA Climate Prediction Center Merged Analysis of Precipitation (CMAP) to approximately define the geographical distribution of surface dryness for the period 1979–2015. This dataset is obtained from five kinds of satellite estimates and is blended with NCEP/NCAR reanalysis values^52^. Although there are some regional discrepancies in magnitudes, trends and inter-annual variabilities of precipitation rates between the CMAP data and several others, we use the annual climatology of precipitation between 1979–2015 to define climate zones, and find similar results among different precipitation data. Given that our results are all based on satellite observations, here only use the CMAP data to define the climate zones in our analyses.

### Short-term satellite-derived datasets

This study uses satellite-derived sounding data made by the Atmospheric Infrared Sounder (AIRS)^53^ to analyze the effects of surface downward longwave radiation (DLR) associated with atmospheric temperature and water vapor changes on LSAT. The AIRS sensor is a cross-track scanning instrument launched on the NASA Aqua satellite in 2002, and hence its products are all available from 2003 onward^54^. The Aqua spacecraft orbits the Earth every 98.8 minutes with an equatorial crossing time at 1:30 P.M. local time (daytime) and 1:30 A.M. local time (nighttime) in a Sun-synchronous, near polar orbit with an inclination of 98.2° and 705 km of operational altitude^55^. Thus, AIRS has a twice-daily temporal resolution with balanced daytime and nighttime measurements in its monthly mean values. AIRS provides not only LSAT and total atmospheric water vapor content (TWV), but also pressure-stratified tropospheric temperatures (T) and specific humidity (*q*). The selected dataset is the version 6 of the AIRS monthly level-3 product at resolution of 1° × 1° grid cells covering the globe. This dataset has passed NASA’s quality control, in which estimated errors of retrieved temperature and water vapor are less than 1 K and 15%, respectively^56, 57^. The prescreening in the AIRS data helps to eliminate retrieved profiles with large errors in the troposphere or near the surface. In addition, high-quality satellite retrievals of surface DLR and its corresponding Cloud Radiative Effect (DLRCRE) as well as surface downward solar radiation (DSR) are also obtained from the Clouds and Earth’s Radiant Energy System (CERES) Energy Balanced and Filled (EBAF) products^58^. This dataset has the same temporal and spatial resolutions as the AIRS datasets.

### Long-term temperature trend analysis

This study reexamines desert amplification from near surface to the upper troposphere using RSS, UAH and GISS datasets. We focus only on the land areas between 50°S–50°N to minimize the effects of polar warming amplification^9, 10, 13^. All the variables are spatially remapped onto a 2.5° × 2.5° grid box using the local area-conservative binning method, and hence in total 1,538 grid boxes are studied. The monthly precipitation is aggregated to generate the annual climatology, and the monthly temperatures are temporally averaged to create annual anomalies based on the 1979–2015 climatology. The linear trend for temperature time series at each grid box is estimated using least squares fitting, and a two-tailed student’s t test is used to tell whether the trend differs significantly from zero. In order to maximize large-scale warming patterns and minimize regional and local scale variabilities, we depict the spatial patterns of temperature trends as a function of climatological precipitation to show how the warming rates vary by large-scale climatic zones as done in *Zhou et al*.^9, 10^. The study region (1,538 grid boxes) is classified into 6, 12, 18 climate zones based on the climatological precipitation values (from the driest to wettest). For each classification, every region contains about the same number of grid boxes except the driest region with more grids as deserts make up ~33% of the global land surface area^13^. The zonal mean time series is generated using area-weighted averaging over the valid grid boxes within each climate zone, and its trend is calculated as done at the grid level. Although more regional and local scale variations are included when more climate zones are classified, the essential features of desert amplification remain robust across all classifications. For simplicity, in most cases we primarily show the annual results for the classification of 12 large-scale climate zones and highlight the distinct features of warming patterns by comparing the results between the driest and wettest from the classified 12 climate zones.

### Short-term anomaly analysis

This study analyzes the relationships among the anomalies in temperatures, DLR and water vapor using satellite products from AIRS and CERES-EBAF datasets for the period 2003–2015. Here we use the 13-year satellite products (2003–2015) as a compromise because there are no reliable long-term observations over land for DLR and water vapor at global scales^1^. Dessler and Davis^29^ showed that the water vapor response to a climate fluctuation at short-term scales should be about the same as that at long-term scales. In this part, we first examine the inter-annual variations of LSAT, DLR and TWV over the driest and wettest from the classified 12 climate zones by precipitation to show the different levels of dependence of LSAT changes on DLR and water vapor. Then we identify the particular significant fluctuation of LSAT in 2010 relative to its climatology (excluding 2010) and examine the overall coupling of LSAT, DLR and TWV anomalies. We further compare the vertical profiles of T and *q* anomalies in 2010 over the driest and wettest climate zones as well as their correlations with DLR and LSAT. These analyses help to assess the relative contributions of atmospheric temperatures and humidity changes to surface warming among different climate zones, and provide further insight into the mechanisms of desert amplification.

## Electronic supplementary material

Supplementary figure
