# Supplementary material for: Observational Evidence for Desert Amplification Using Multiple Satellite Datasets
Source: Sci Rep. 2017 May 17;7:2043. doi: 10.1038/s41598-017-02064-w (PMC5435705; doi:10.1038/s41598-017-02064-w)
Supplement: Supplementary file 1 — Supplementary figure [file 41598_2017_2064_MOESM1_ESM.pdf]

Supporting Figure

Observational Evidence for Desert Amplification Using Multiple Satellite Datasets

Nan Wei[1,2], Liming Zhou[2]*, Yongjiu Dai[1], Geng Xia[2], Wenjian Hua[2,3]

[1]School of Atmospheric Sciences, Sun Yat-Sen University, Guangzhou, 519082, China

[2]Department of Atmospheric and Environmental Sciences, University at Albany, State University of New York, Albany, NY 12222, USA

[3]Key Laboratory of Meteorological Disaster, Ministry of Education (KLME)/Joint International Research Laboratory of Climate and Environment Change (ILCEC)/Collaborative Innovation Center on Forecast and Evaluation of Meteorological Disasters (CIC-FEMD), Nanjing University of Information Science & Technology, Nanjing, 210044, China

List of supporting figure

Supporting Figure S1

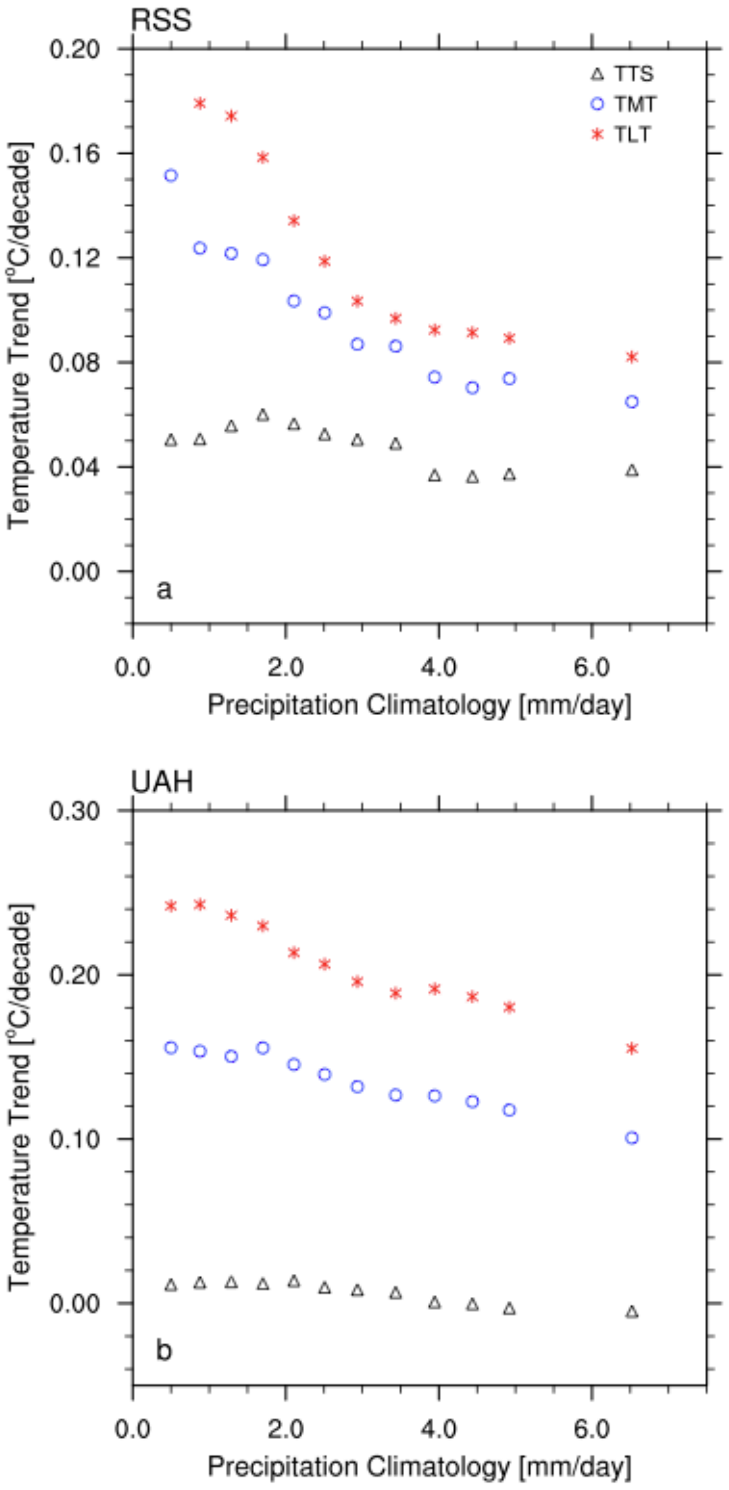

Figure S1. Zonal mean annual temperature trends (°C/decade) of TLT, TMT and TTS from (a) RSS and (b) UAH for the period 1979–2015 as a function of zonal mean climatological annual precipitation (mm/day) in terms of 12 large-scale climate zones.
